# Hydrogen-oxygen mixture inhalation as an adjunctive treatment to home-based exercise in older patients with knee osteoarthritis: an open-label, blinded-endpoint, randomized controlled trial

**DOI:** 10.3389/fphar.2025.1505922

**Published:** 2025-01-30

**Authors:** Chenhui Wang, Mengwei Yan, Yuru Li, Lei Han, Hongqian Wang, Shufeng Jia, Xingchen Liu, Yang Liu, Fan Wu, Baoguo Wang

**Affiliations:** ^1^ Department of Anesthesiology, Sanbo Brain Hospital, Capital Medical University, Beijing, China; ^2^ Department of Pulmonary and Critical Care Medicine, Center of Respiratory Medicine, China-Japan Friendship Hospital, Capital Medical University, Beijing, China; ^3^ Infirmary, Taikang Yanyuan Continuing Care Retirement Community, Beijing, China; ^4^ School of Nursing, Harbin Medical University, Harbin, Heilongjiang, China

**Keywords:** molecular hydrogen, elderly, osteoarthritis, WOMAC, exercise therapy

## Abstract

**Objective:**

Knee osteoarthritis (KOA) is a degenerative joint condition, leading to disability and diminished quality of life. Molecular hydrogen has been proven to have antioxidant and anti-inflammatory properties, but few studies have investigated its effects on osteoarthritis. Our study aims to assess the therapeutic potential of hydrogen-oxygen mixture (H_2_-O_2_) inhalation for KOA.

**Methods:**

In this randomized controlled trial, eligible elderly KOA patients were randomly assigned to either Group H or Group C. Both groups participated in a 12-week home-based exercise (HBE) program, which included knee-joint exercises and health education. Group H additionally received H_2_-O_2_ inhalation for 60 min per day over 2 weeks, while Group C did not. The primary outcome was measured using Western Ontario and McMaster Universities Osteoarthritis Index (WOMAC). Secondary outcomes included inflammation levels (hs-CRP, NLR, PLR, LMR), Chair Stand Test (CST), Timed Up and Go (TUG), 36-item short-form health survey (SF-36), Exercise Adherence Rating Scale (EARS), and adverse events.

**Results:**

A total of 121 subjects were enrolled, with an average age of 81.2 years, and 80.2% were female. The between-group mean difference in the WOMAC total score was −5.2 (95% CI −12.1 to 1.7, P = 0.140) at week 12, with Group H showing an improvement of −22.9 (95% CI −26.3 to −19.6, P < 0.001) and Group C showing an improvement of −19.4 (95% CI −22.7 to −16.0, P < 0.001) compared to baseline, revealing a significant group × time interaction (F (3, 356.034) = 14.425, P < 0.001). No significant differences were observed between both groups at week 12 in CST, TUG, SF-36 scores, EARS scores, or the incidence of adverse events.

**Conclusion:**

Although clinical significance was not achieved, H_2_-O_2_ inhalation alleviated KOA symptoms and enhanced functional activity in elderly patients undergoing the HBE program during the initial 2 weeks. However, its sustained effects on improving KOA symptoms were not observed.

## 1 Introduction

Knee osteoarthritis (KOA) is a common degenerative joint condition impacting the cartilage in the knee joint and surrounding tissues, stemming from various factors such as mechanical stress, inflammation, and metabolic dysregulation (Katz et al., 2021). Its primary clinical symptoms consist of pain in the joints, swelling, stiffness, limited joint function, and potentially even functional loss (Johnson and Hunter, 2014; Liu et al., 2015). It is estimated that KOA affects approximately 21%–33% of elderly individuals in China, with its onset typically manifesting gradually throughout aging (Sun et al., 2019). Notably, KOA can lead to adverse consequences, including disability and diminished quality of life (QoL), ultimately resulting in a substantial socio-economic burden (Sun et al., 2019; Hunter and Bierma-Zeinstra, 2019).

In accordance with clinical guidelines, exercise therapy stands out as a pivotal treatment for KOA, capable of retarding disease progression, alleviating pain, and improving knee function (Huang et al., 2018; Kolasinski et al., 2019). To date, more scholars have recognized the value of home-based knee exercises and relevant studies have confirmed the efficacy and feasibility of home-based knee exercises for alleviating pain and improving function among KOA patients (Chen H. et al., 2019; Safran-Norton et al., 2019). Nevertheless, most of the interventions of home-based exercises varied among different studies and there are few comprehensive interventions encompassing both knee exercises and lifestyle education available in China. Initiative Practice for Health (IPFH) is a comprehensive intervention strategy aimed at encouraging health-promoting behaviors related to balanced diet, physical activity, social engagement, and mental wellbeing, with the goal of fostering healthier lifestyles (Zhang et al., 2022). Based on the concept of IPFH, we designed a home-based exercise (HBE) program for KOA patients, which includes knee-joint exercises and health education about lifestyle behaviors. Although our preliminary study demonstrated the efficacy of the HBE program in improving KOA-related symptoms and enhancing QoL (Zhang, 2023), symptom improvement was not often satisfactory in elderly KOA patients, which may be attributed to the decline in their physical function and activity ability. Hence, the development of safe and effective adjuvant therapies for symptom management in older patients with KOA is essential.

It is acknowledged that the onset and progression of KOA are primarily attributed to the degeneration and damage of articular cartilage and the resultant chronic inflammation reactions (Lv et al., 2021; Shumnalieva et al., 2023). Evidence suggested that exercise therapy alleviated pain and improved functional symptoms associated with KOA by reducing oxidative stress, accelerating the restoration of impaired cartilage, and decreasing levels of inflammatory factors (Fransen et al., 2015; Wellsandt and Golightly, 2018). Interestingly, molecular hydrogen has also been proven to have antioxidant and anti-inflammatory functions (Ishibashi, 2013). Cell and animal studies have shown that hydrogen-rich water or the administration of hydrogen-releasing hydrogel effectively mitigates osteoarthritis-induced cartilage damage, promotes cartilage regeneration, inhibits cell apoptosis, and accelerates cartilage matrix repair (Zhang et al., 2023; Cheng et al., 2020a). These benefits are likely associated with the suppression of the JNK signaling pathway and the downregulation of Wnt/β-catenin activation in chondrocytes (Lu et al., 2021; Lin et al., 2016). However, the effect of molecular hydrogen on clinical outcomes (pain, stiffness, and functional status) in osteoarthritis conditions remains unclear at present and there have been no relevant clinical trials reported in this area.

Therefore, our study seeks to assess the therapeutic potential of hydrogen molecules in the treatment of KOA. We hypothesized that hydrogen-oxygen (H_2_-O_2_) mixture inhalation could improve KOA-related symptoms, such as pain, stiffness, and function, compared to those who did not receive the H_2_-O_2_ mixture inhalation in elderly KOA patients undergoing the HBE program.

## 2 Materials and methods

### 2.1 Study design and participants

From July 2023 to February 2024, at Taikang Yanyuan Continuing Care Retirement Community (CCRC) in China, an open-label, blinded-endpoint, randomized controlled trial was conducted. The recruitment process in this CCRC was conducted via roll-up banners and internal social media advertisements. The Ethics Committee of Sanbo Brain Hospital, Capital Medical University, approved the study (SBNK-YJ-2023-014-01), and it was registered with the Chinese Clinical Trial Registry (ChiCTR2300073102). Participants or their legally authorized representatives provided informed consent prior to enrollment.

Participants were required to meet the following criteria for inclusion: 1) age ≥65 years. 2) diagnosis of KOA identified from Taikang healthcare medical records. 3) Kellgren-Lawrence (KL) grade was classified as level 2 or 3. 4) duration of KOA ≥6 months and did not receive joint replacement surgery or arthroscopic surgery. 5) self-reported knee-joint pain ≥4 on the numeric rating scale (NRS) during the past week. Exclusion criteria for participants encompassed the following conditions: 1) mental disorders; 2) cognitive impairment; 3) severe systematic diseases such as severe cardiopulmonary disease or multi-organ failure; 4) inability to cooperate in completing exercises; 5) intolerance to inhalation therapy; 6) involvement in other clinical trials within the previous 3 months.

### 2.2 Randomization and blinding

Participants eligible for the study were randomly allocated to either the H_2_-O_2_ mixture inhalation group (Group H) or the control group (Group C) in a 1:1 ratio. Randomization, performed by a researcher not engaged in the study, utilized block randomization with block sizes of 4 and 6. Randomization codes were enclosed in opaque sealed envelopes sequentially numbered, and then revealed by a researcher in sequence post-recruitment. Participants were informed of their randomization assignment after completing baseline assessments. Only the outcome assessors were blind to group allocation and treatment.

### 2.3 Procedures

All included participants would undergo a 12-week HBE program, which mainly included health education on lifestyle behaviours for KOA and home-based exercise therapy. All of the participants were required to attend health lectures held in the CCRC at week 4 and 8. Health education included 4 modules covering the concepts of clinical manifestations and risk factors, strategies for managing knee-joint pain, dietary recommendations, and exercise guidance for KOA. Following the lecture, each participant would receive a printed copy of the material outlining the HBE guidance manual for KOA. Moreover, the study assistants would assist participants in logging into our Northern Smart Platform on their mobile phones. Here, they can access home-based knee exercise videos and health knowledge short videos, enhancing their comprehension and retention of health and exercises.

**FIGURE 1:**
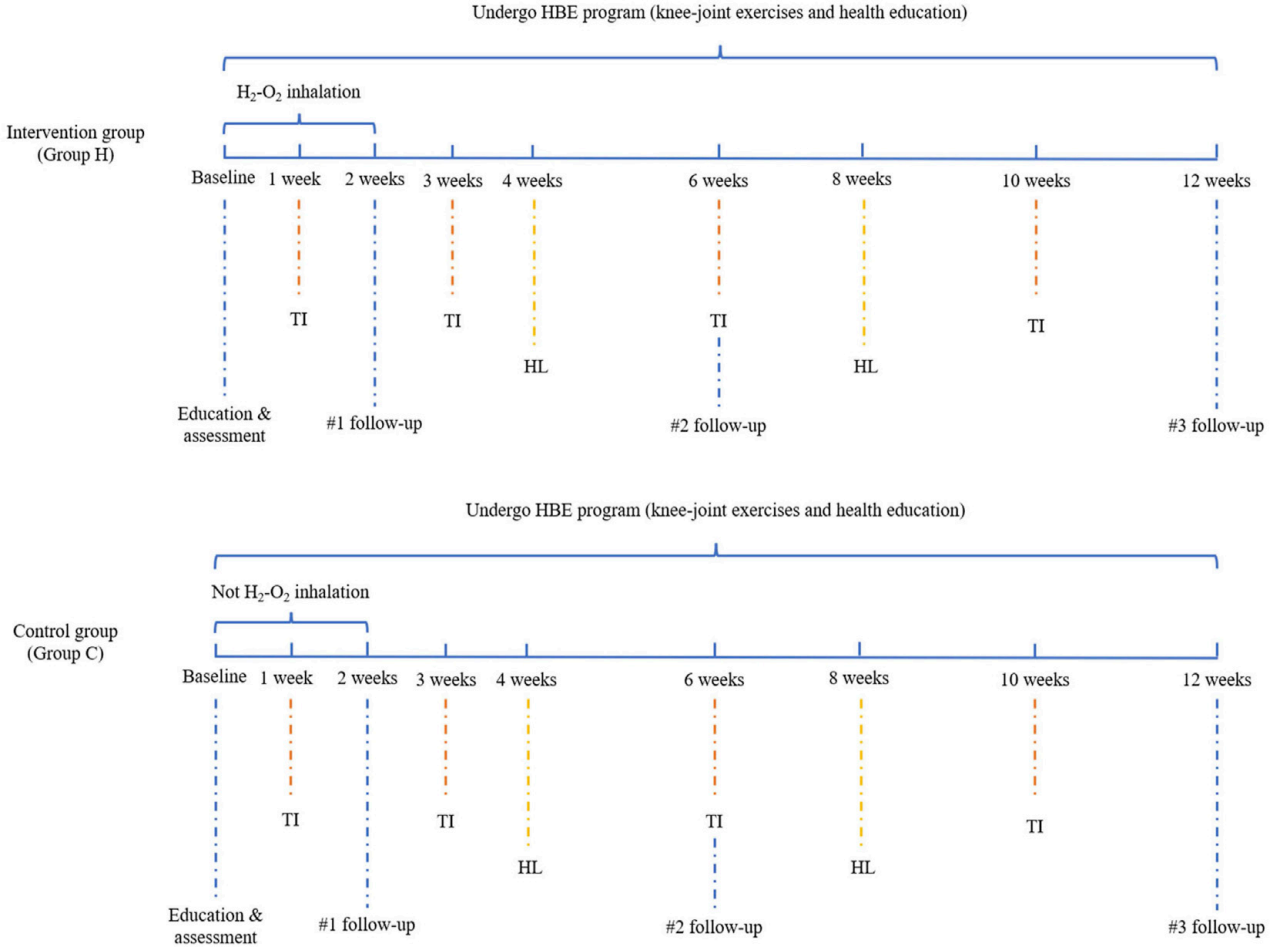
Flow chart of H2-O2 inhalation and HBE program. Abbreviation: TI, telephone interview; HL, health lectures.

Knee-joint exercise therapy was formulated based on a synthesis of literature review, clinical practice, and multidisciplinary expert group discussions. The primary objectives were to bolster lower-limb muscle strength, improve balance, reduce pain, and alleviate knee stiffness. The regimen encompassed warm-up exercises, passive knee flexion and extension, isometric quadriceps contractions, supine straight-leg lifts, prone leg lifts, resistance knee extension and flexion (details in Supplementary File 1). All participants were required to perform the above home-based exercises during the study at least 3 days a week, for 45–60 min a day, during 12 weeks. The initial knee exercises were guided by a specialized pain physician and physical therapist, while subsequent home-based knee exercises were performed independently, without supervision. Additionally, participants were asked to record their daily completion status of home-based exercises. Upon successful completion of the 12-week home-based exercise regimen, participants would be rewarded with a gift as an incentive to maintain their commitment to home-based exercises. Meanwhile, telephone interviews were conducted with participants during weeks 1, 3, 6, and 10 to evaluate their exercise advancement and to motivate adherence to the exercise regimen (Figure 1).

### 2.4 Intervention and control

Participants in Group H received H_2_-O_2_ mixture inhalation utilizing nasal tubes and the hydrogen-oxygen nebulizer AMS-H-01 (manufactured by Shanghai Asclepius Meditec Co., Ltd., China), delivering 2.0 L/min of hydrogen and 1.0 L/min of oxygen. Daily inhalation sessions lasted for 60 min continuously over a period of 2 weeks. The basis of our intervention regimen was provided by the published research about H_2_-O_2_ inhalation in alleviating postherpetic neuralgia (Wang et al., 2016). Conversely, participants in Group C did not undergo H_2_-O_2_ mixture inhalation therapy.

### 2.5 Outcomes measurement

The primary outcomes of the study included pain, joint stiffness, and functional status associated with KOA, evaluated through the Western Ontario and McMaster Universities Osteoarthritis Index (WOMAC) (Bellamy et al., 1988). This index consisted of 24 items: 5 for pain assessment, 2 for joint stiffness, and 17 for joint function. Each item was evaluated using the Visual Analog Scale (VAS) method, with scores ranging from 0 to 10 points (Heller et al., 2016). Higher scores denoted greater severity of pain, joint stiffness, or limitations in joint function. The WOMAC scale we used was the validated Chinese version with good internal reliability and test-retest reliability (Xie et al., 2008).

The secondary outcomes of the study included inflammation levels, measured by the neutrophil-lymphocyte ratio (NLR), platelet-lymphocyte ratio (PLR), lymphocyte-monocyte ratio (LMR), and high-sensitivity C-reactive protein (hs-CRP), as well as other objective evaluation such as the chair stand test (CST), timed up and go (TUG), and QoL. The increase in hs-CRP, NLR, and PLR levels, along with a decrease in LMR levels, suggests an exacerbation of the inflammatory response. During the CST, participants were seated in a 45 cm-high armless chair, maintaining a shoulder-width stance with arms crossed over their chests. They were directed to perform as many sit-to-stand cycles as possible within a 30-s timeframe (Jones et al., 1999). For the TUG test, participants were instructed to rise from a chair, walk a distance of 3 m, turn around, return to the starting position, and sit back down (Sabirli et al., 2013). Both the CST and TUG were conducted twice, with a 5-min rest period in between to minimize fatigue. The maximum number of completions in the CST and the total duration of the TUG test in seconds were recorded. QoL was measured utilizing the 36-item short-form health survey (SF-36) (Brazier et al., 1992). This scale evaluates 8 dimensions of health: Physical Function (PF), Bodily Pain (BP), Role Limitations due to Physical Health Problems (RP), Role Limitations due to Emotional Problems (RE), General Mental Health (MH), Social Function (SF), Vitality (VIT), and General Health (GH). Higher scores indicate a better health status. Adherence to home-based exercises was assessed using the Exercise Adherence Rating Scale (EARS), which encompasses a scoring range of 0–24 points (Newman-Beinart et al., 2017). A higher score on the EARS indicates better adherence to the exercise regimen.

### 2.6 Data collection and follow-up

Baseline data, including age, sex, body mass index (BMI), education levels, affected side, duration of KOA, analgesic usage, and medical history, were gathered through the Taikang healthcare electronic system. KL grade was assessed using X-ray imaging of the knee joints (Liew et al., 2023). NRS of knee-joint pain, baseline of WOMAC, CST, TUG, and SF-36 were evaluated during the enrollment evaluation. NLR, PLR, LMR, and hs-CRP were tested at baseline and week 12.

Participants were scheduled for follow-up at weeks 2, 6, and 12 after enrollment. During these follow-up interviews, WOMAC, CST, and TUG were assessed at weeks 2, 6, and 12. SF-36 and adherence assessment were conducted at week 12. Additionally, any adverse events occurring during the trial were recorded.

### 2.7 Sample size calculation

Sample size calculation was predicated on a prior randomized controlled trial, where the standard deviation (SD) of the exercise intervention group for KOA patients was noted as 15.2 (Chao et al., 2021). The minimal clinically important difference (MCID) in total WOMAC score, as calculated in various studies, ranged from 9 to 22 points (Bhandari et al., 2019). We hypothesized that the combination of H_2_-O_2_ mixture inhalation with HBE could reduce the WOMAC score of KOA patients by 9 points compared to the control group only receiving HBE. Therefore, we aimed to enroll 46 patients in each group, aiming for 80% power at a two-sided significance level of 5%. Accounting for an anticipated dropout rate of 20%, we aimed to recruit a minimum of 116 patients for this study.

### 2.8 Statistical analysis

The normality of quantitative variables was assessed using the Kolmogorov-Smirnov test. Continuous data were expressed as medians and interquartile ranges (IQRs) or as mean and SD, depending on their distribution. Categorical data were presented as frequency distributions. Analysis of differences was performed using the Mann-Whitney U test, Student’s t-test, or chi-square test, based on the data type and distribution. A linear mixed-effects model was employed to analyze repeated measurement data including WOMAC and WOMAC subscale scores, CST, and TUG. Fixed effects included treatment group, time effect, and a treatment × time interaction. The significance of the treatment × time interaction indicated a significant treatment effect. Random effects encompassed time, where both intercept and slope were allowed to vary. Post hoc exploratory analyses were conducted to evaluate the heterogeneity of the primary outcome across predefined subgroups, including age, sex, KL grade, EARS score, and duration of KOA. Treatment-by-covariate interactions for each subgroup factor were examined separately using a general linear model. The outcomes of repeated measurement data were represented by mean and 95% confidence interval (CI). Intention-to-treat (ITT) analysis was regarded as the primary analysis, with per-protocol analysis considered a sensitivity analysis to verify the robustness of the outcomes. Missing data were imputed using mean imputation in cases where missing values accounted for less than 20% of the data. If missing data exceeded 20%, no imputation was applied and analyses were restricted to the available data.

All statistical analyses were conducted using SPSS (version 23.0, IBM, USA) and GraphPad Prism (version 8.0, GraphPad, United States). Two-sided statistical tests were employed, and statistical significance was established at a value of P < 0.05.

## 3 Results

**FIGURE 2:**
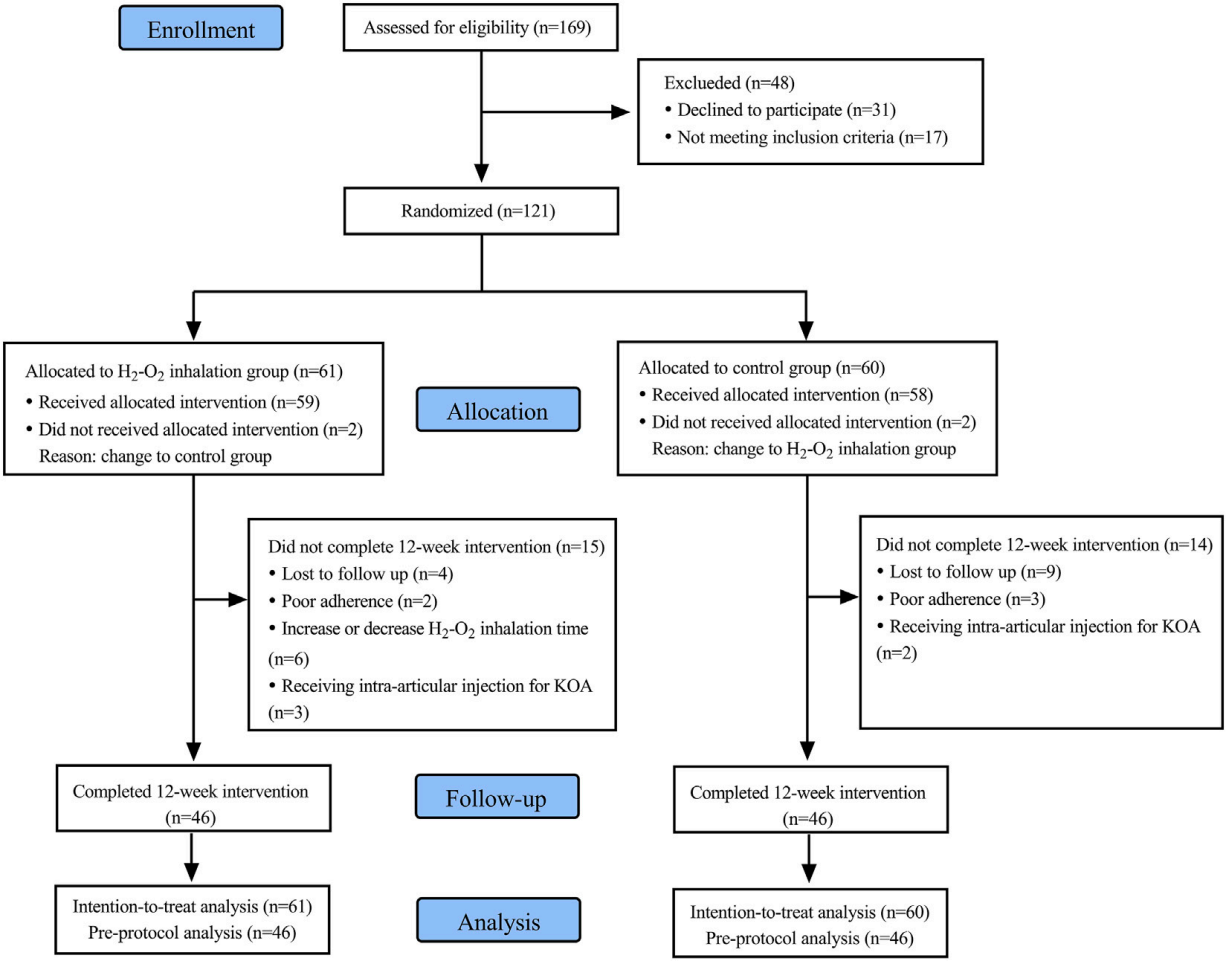
Flow chart of subject inclusion.

Out of the initial 169 subjects screened for this study, 17 did not meet the inclusion criteria, and 31 declined to participate. Ultimately, 121 subjects were enrolled, with 61 assigned to Group H and 60 to Group C. However, 2 subjects from each group did not receive the assigned intervention because of their preference to switch to the other group. Additionally, 15 subjects in Group H and 14 subjects in Group C discontinued the study. In Group H, 4 were lost to follow-up, 2 showed poor adherences, 6 had increased or decreased H_2_-O_2_ inhalation time, and 3 received intra-articular injections. In Group C, 9 were lost to follow-up, 3 exhibited poor adherences, and 2 received intra-articular injections. Finally, 46 subjects in each Group completed the 12-week study period (Figure 2).

### 3.1 Baseline characteristics

Most of the individuals enrolled in our study were female (80.2%, 97/121), with a mean age of 81.2 ± 6.1 years. Bilateral KOA was the most prevalent condition, impacting 66.9% (81/121) of participants, while KL grade 3 was the most common classification, affecting 52.9% (64/121) of the subjects. The median duration of KOA was 20 (10–30) years. The mean NRS of knee-joint pain was 4.4 ± 0.7. No significant difference was observed in demographic characteristics and medical comorbidities between the two groups (Table 1). Similar results were also obtained through pre-protocol analysis (Supplementary Table S1).

**TABLE 1:** ITT analysis of demographic and clinical characteristics of participants by arm in baseline.

Variables	Total (n = 121)	Group H (n = 61)	Group C (n = 60)	df	t, Z or chi-square	P
Age, years, mean (SD)	81.2 (6.1)	80.1 (5.7)	82.2 (6.4)	119	−1.899	0.060
Sex, female, n (%)	97 (80.2)	49 (80.3)	48 (80.0)	1	0.018	0.895
BMI, kg/m^2^, mean (SD)	23.7 (3.0)	23.6 (2.9)	23.8 (3.1)	119	−0.224	0.823
Affected side, n (%)				1	1.200	0.273
Unilateral	40 (33.1)	23 (37.7)	17 (28.3)			
Bilateral	81 (66.9)	38 (62.3)	43 (71.7)			
KL grade, n (%)				1	0.212	0.645
2	57 (47.1)	30 (49.2)	27 (45.0)			
3	64 (52.9)	31 (50.8)	33 (55.0)			
NRS for the past week, mean (SD)	4.4 (0.7)	4.4 (0.6)	4.3 (0.7)	119	0.229	0.820
Duration of KOA, years, median (IQR)	20 (10-30)	20 (9–26.5)	20 (10-30)	119	−0.764	0.445
Education level, n (%)				2	0.637	0.727
High school education or less	19 (15.7)	8 (13.1)	11 (18.3)			
Associate degree	36 (29.8)	19 (31.1)	17 (28.3)			
Bachelor degree or above	66 (54.6)	34 (55.7)	32 (53.3)			
Cigarette smoking, n (%)	4 (3.3)	2 (3.3)	2 (3.3)	1	<0.001	1.000
Alcohol drinking, n (%)	9 (7.4)	4 (6.5)	5 (8.3)	1	0.139	0.743
Medical history, n (%)						
Respiratory disease	33 (27.3)	16 (26.2)	17 (28.3)	1	0.067	0.795
Hypertension	88 (72.7)	45 (73.8)	43 (71.7)	1	0.067	0.795
Coronary heart disease	53 (43.8)	25 (41.0)	28 (46.7)	1	0.397	0.529
Gastrointestinal disease	32 (26.4)	17 (27.9)	15 (25.0)	1	0.128	0.721
Diabetes mellitus	37 (30.6)	18 (29.5)	19 (31.7)	1	0.066	0.797
Anemia	1 (0.8)	1 (1.6)	0 (0)	-	-	-
Chronic kidney disease	4 (3.3)	1 (1.6)	3 (5.0)	1	1.069	0.365
Cancer	10 (8.3)	6 (9.8)	4 (6.7)	1	0.401	0.743
Cerebrovascular disease	27 (22.3)	12 (19.7)	15 (25.0)	1	0.495	0.482
Other	29 (24.0)	14 (23.0)	15 (25.0)	1	0.070	0.792
Analgesics taken, n (%)	73 (60.3)	39 (63.9)	34 (56.7)	1	0.668	0.414
Types of analgesics taken, n (%)				1	0.214	0.644
NSAIDS	45 (61.6)	25 (64.1)	20 (58.8)			
Chinese medicine plaster	28 (38.4)	14 (36.8)	14 (41.2)			

Data are expressed as n (%), mean (SD) or median (IQR). df = degree of freedom; SD, standard deviation; BMI, body mass index; KOA, knee osteoarthritis; IQR, interquartile range; NSAIDS, nonsteroidal anti-inflammatory drugs.

### 3.2 Pain intensity, stiffness and function of KOA

**FIGURE 3:**
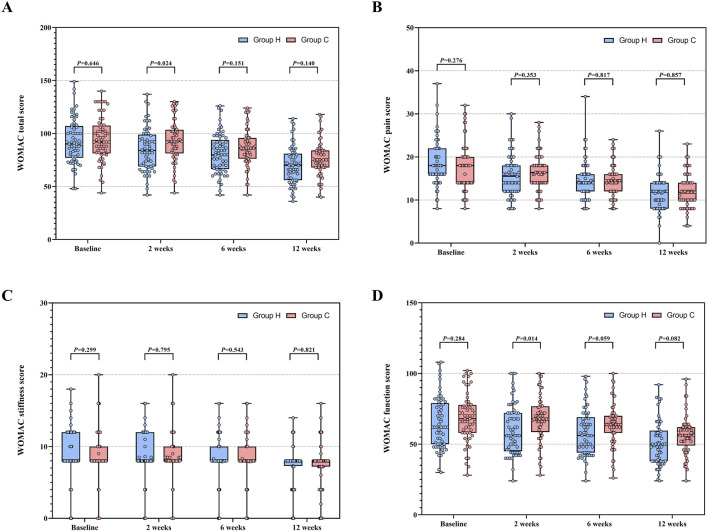
WOMAC total score and its subscale scores between Group H and Group C during the study period. **(A)** Distribution of WOMAC total score. **(B)** Distribution of WOMAC pain score. **(C)** Distribution of WOMAC stiffness score. **(D)** Distribution of WOMAC function score. Abbreviation: WOMAC, Western Ontario and McMaster Universities Osteoarthritis Index.

**FIGURE 4:**
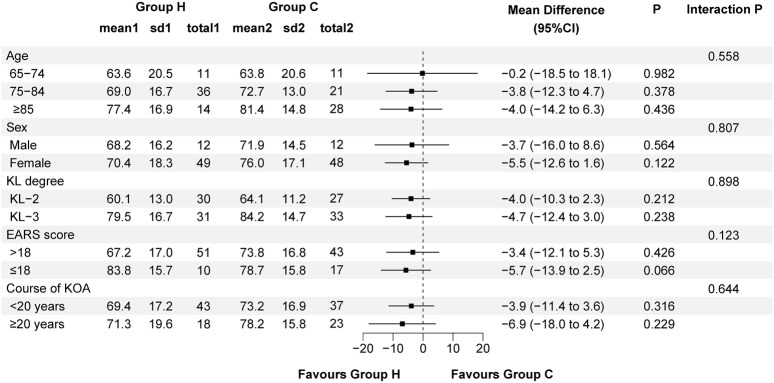
Forest plot assessing the improvement of WOMAC total score between Group H and Group C in predefined subgroups. Abbreviation: KL, Kellgren-Lawrence; EARS, Exercise Adherence Rating Scale; KOA, knee osteoarthritis.

Data distribution and changes in WOMAC total score and its subscale scores are detailed in Table 2 and Figure 3. In ITT analysis, the linear mixed-effects model revealed the between-group mean difference in the WOMAC total score of −5.2 (95% CI -12.1 to 1.7, F (1, 135.411) = 2.206, P = 0.140) at week 12, with Group H showing an improvement of −22.9 (95% CI −26.3 to −19.6, F (3, 356.034) = 128.131, P < 0.001) and Group C showing an improvement of −19.4 (95% CI −22.7 to −16.0, F (3, 356.034) = 100.426, P < 0.001) compared to baseline. Notably, the mean difference in the WOMAC total score peaked at week 2 (MD: −8.0, 95% CI -14.9 to −1.1, F (1, 135.411) = 5.229, P = 0.024), indicating distinct declining trends. Additionally, the between-group mean difference in WOMAC pain score and function score was −0.2 (95% CI -1.9 to 1.5, F (1,169.895) = 0.032, P = 0.858) and −5.2 (95% CI -11.1 to 0.7, F (1, 135.326) = 3.070, P = 0.082) at week 12 respectively, with improvements of −6.9 (95% CI −8.3 to −5.5, F (3, 349.291) = 66.295, P < 0.001) and −14.2 (95% CI −17.0 to −11.4, F (3, 356.451) = 71.845, P < 0.001) in Group H, and −5.8 (95% CI −7.2 to −4.4, F (3, 349.291) = 43.290, P < 0.001) and −12.2 (95% CI −15.0 to −9.4, F (3, 356.451) = 64.406, P < 0.001) in Group C compared to baseline. Similarly, the mean difference in the WOMAC pain score (MD: −0.8, 95% CI -2.5 to 0.9, F (1, 169.895) = 0.866, P = 0.353) and function score (MD: −7.4, 95% CI -13.3 to −1.6, F (1, 135.326) = 6.254, P = 0.014) also reached their apex at week 2, indicating varied patterns of decline. Significant group × time interactions were observed for the WOMAC total score (F (3, 356.034) = 14.425, P < 0.001), pain score (F (3, 349.291) = 6.218, P < 0.001), and function score (F (3, 356.451) = 8.870, P < 0.001) between the two groups, while no significant group × time interactions were noted for the WOMAC stiffness score (F (3, 355.959) = 1.544, P = 0.230). Similar results were also obtained through pre-protocol analysis (Supplementary Table S2). In subgroup analysis, there were no significant interactions between treatment group and predefined factors, such as age, sex, KL grade, EARS score, and duration of KOA (Figure 4).

**TABLE 2:** ITT analysis of WOMAC scores, CST and TUG of participants by arm in follow-ups.

	H Group (n = 61)	C Group (n = 60)	Mean difference (95% CI)	Numerator df	Denominator df	F	P
WOMAC Total Score, mean (95% CI)							
Baseline	92.9 (80.0, 97.8)	94.5 (89.6, 99.4)	−1.6 (−8.5, 5.3)	1	135.441	0.212	0.646
2 weeks	84.5 (79.6, 89.4)	92.5 (87.6, 97.5)	−8.0 (−14.9, −1.1)	1	135.441	5.229	0.024
6 weeks	81.2 (76.3, 86.1)	86.3 (81.4, 91.2)	−5.1 (−12.0, 1.9)	1	135.441	2.088	0.151
12 weeks	69.9 (65.1, 74.8)	75.2 (70.2, 80.1)	−5.2 (−12.1, 1.7)	1	135.441	2.206	0.140
Pain Score, mean (95% CI)							
Baseline	18.5 (17.3, 19.7)	17.6 (16.4, 18.8)	0.9 (−2.6, 0.8)	1	169.895	1.195	0.276
2 weeks	15.6 (14.4, 16.8)	16.4 (15.2, 17.6)	−0.8 (−2.5, −0.9)	1	169.895	0.866	0.353
6 weeks	14.7 (13.5, 15.9)	14.5 (13.3, 15.7)	0.2 (−1.5, 1.9)	1	169.895	0.054	0.817
12 weeks	11.6 (10.5, 12.8)	11.8 (10.6, 13.0)	−0.2 (−1.9, 1.5)	1	169.895	0.032	0.857
Stiffness Score, mean (95% CI)							
Baseline	9.2 (8.3, 10.0)	8.6 (7.7, 9.4)	0.6 (−1.8, 0.6)	1	155.504	1.086	0.299
2 weeks	8.6 (7.8, 9.5)	8.5 (7.6, 9.3)	0.2 (−1.0, 1.4)	1	155.504	0.068	0.795
6 weeks	8.3 (7.5, 9.2)	7.9 (7.1, 8.8)	0.4 (−0.8, 1.6)	1	155.504	0.372	0.543
12 weeks	7.3 (6.5, 8.2)	7.2 (6.3, 8.0)	0.1 (−1.1, 1.3)	1	155.504	0.051	0.821
Function Score, mean (95% CI)							
Baseline	65.2 (61.1, 69.3)	68.4 (64.2, 72.5)	−3.2 (−9.0, 2.7)	1	135.326	1.156	0.284
2 weeks	60.3 (56.2, 64.4)	67.7 (63.5, 71.9)	−7.4 (−13.3, −1.6)	1	135.326	6.254	0.014
6 weeks	58.2 (54.1, 62.3)	63.8 (59.7, 68.0)	−5.6 (−11.5, 0.2)	1	135.326	3.618	0.059
12 weeks	51.0 (46.8, 55.1)	56.2 (52.0, 60.3)	−5.2 (−11.1, 0.7)	1	135.326	3.070	0.082
Chair Stand Test (CST), mean (95% CI)							
Baseline	8.4 (7.6, 9.2)	7.7 (6.9, 8.5)	0.7 (−0.5, 1.8)	1	146.764	1.405	0.238
2 weeks	8.8 (8.0, 9.6)	7.9 (7.1, 8.7)	0.9 (−0.3, 2.0)	1	146.764	2.497	0.116
6 weeks	9.9 (9.1, 10.7)	9.3 (8.5, 10.1)	0.6 (−0.5, 1.7)	1	146.764	1.115	0.293
12 weeks	11.4 (10.6, 12.2)	10.5 (9.7, 11.3)	0.9 (−0.3, 1.9)	1	146.764	2.264	0.135
Timed Up and Go (TUG), mean (95% CI)							
Baseline	14.2 (12.8, 15.6)	15.5 (14.1, 16.9)	−1.4 (−3.4, 0.7)	1	134.075	1.795	0.183
2 weeks	13.5 (12.1, 14.9)	15.1 (13.7, 16.6)	−1.6 (−3.6, 0.4)	1	134.075	2.484	0.117
6 weeks	12.8 (11.4, 14.2)	13.9 (12.6, 15.4)	−1.2 (−3.2, 0.8)	1	134.075	1.353	0.247
12 weeks	12.0 (10.6, 13.4)	13.1 (11.7, 14.5)	−1.1 (−3.1, 0.9)	1	134.075	1.134	0.289

Data are expressed as mean (95% CI). df = degree of freedom; WOMAC, Western Ontario and McMaster Universities Osteoarthritis Index; CST, chair stand test; TUG, timed up and go; CI, confidence interval.

Notes: WOMAC, total score: group× time interaction, F (3, 356.034) = 14.425, P < 0.001. WOMAC, pain score: group× time interaction, F (3, 349.291) = 6.218, P < 0.001. WOMAC, stiffness score: group× time interaction, F (3, 355.959) = 1.544, P = 0.203. WOMAC, function score: group× time interaction, F (3, 356.451) = 8.870, P < 0.001. CST: group× time interaction, F (3, 354.157) = 1.299, P = 0.274. TUG: group× time interaction, F (3, 355.671) = 0.891, P = 0.446.

### 3.3 Chair stand test and timed up and go

**FIGURE 5:**
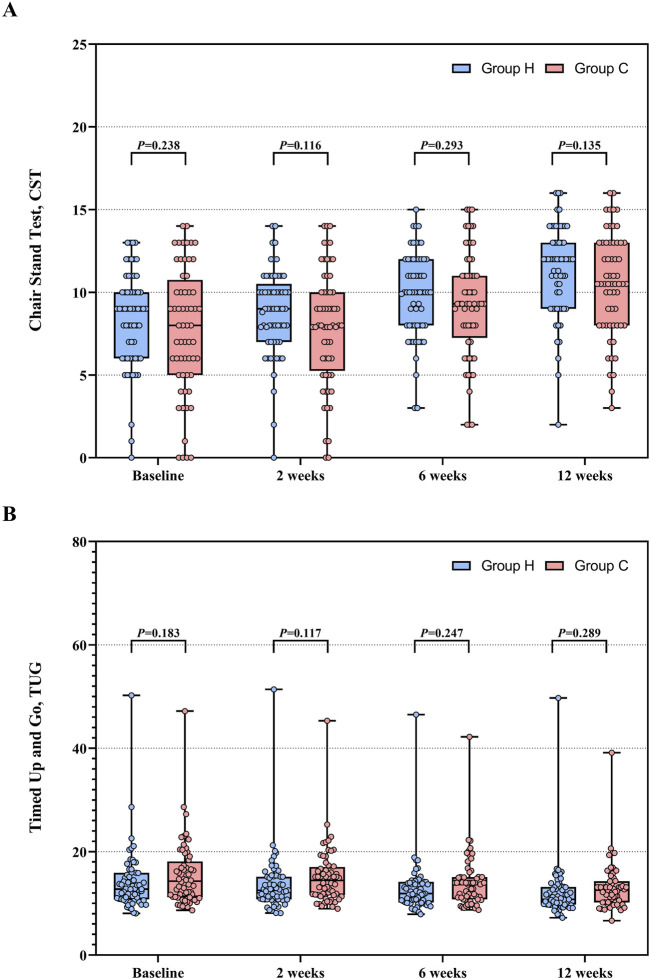
CST and TUG between Group H and Group C during the study period. **(A)** Distribution of CST. **(B)** Distribution of TUG. Abbreviation: CST, chair stand test; TUG, timed up and go.

Baseline data for CST and TUG were comparable in both groups. The between-group mean difference in CST increased from 0.7 (95%CI −0.5 to 1.8, F (1, 146.764) = 1.405, P = 0.238) to 0.9 (95%CI −0.3 to 1.9, F (1, 146.764) = 2.264, P = 0.135), and TUG decreased from −1.4 (95%CI −3.4 to 0.7, F (1, 134.075) = 1.795, P = 0.183) to −1.1 (95%CI -3.1 to 0.9, F (1, 134.075) = 1.134, P = 0.289). For CST, Group H exhibited a significant increase at week 12 compared to baseline (MD: −3.0, 95% CI: −3.7 to −2.3; F (3, 354.157) = 48.769, P < 0.001). Similarly, Group C also showed a significant increase in CST at week 12 (MD: −2.8, 95% CI: −3.5 to −2.1; F (3, 354.157) = 46.245, P < 0.001). For TUG, Group H demonstrated a significant reduction at week 12 compared to baseline (MD: 2.1, 95% CI: 1.2 to 3.1; F (3, 355.671) = 12.027, P < 0.001). Similarly, Group C showed a significant reduction in TUG at week 12 compared to baseline (MD: 2.4, 95% CI: 1.5 to 3.4; F (3, 355.671) = 17.069, P < 0.001). Based on a linear mixed-effects model, ITT analysis demonstrated a significant increasing trend in CST (time effect: F (3, 354.157) = 93.694, P < 0.001) and a significant decreasing trend in TUG (time effect: F (3, 355.671) = 28.247, P < 0.001) throughout the study period in both groups. However, no group effects were observed in either CST (group effect: F (1, 121.740) = 1.957, P = 0.164) or TUG (group effect: F (1, 120.595) = 1.747, P = 0.189) between the two groups. Group × time interactions were not significant in CST (F (3, 354.157) = 1.299, P = 0.274) and TUG (F (3, 355.671) = 0.891, P = 0.446) between the two groups. Data distribution and changes in CST and TUG are presented in Table 2 and Figure 5. Pre-protocol analysis yielded similar findings (Supplementary Table S2).

### 3.4 Inflammation levels (hs-CRP, NLR, PLR, and LMR)

**FIGURE 6:**
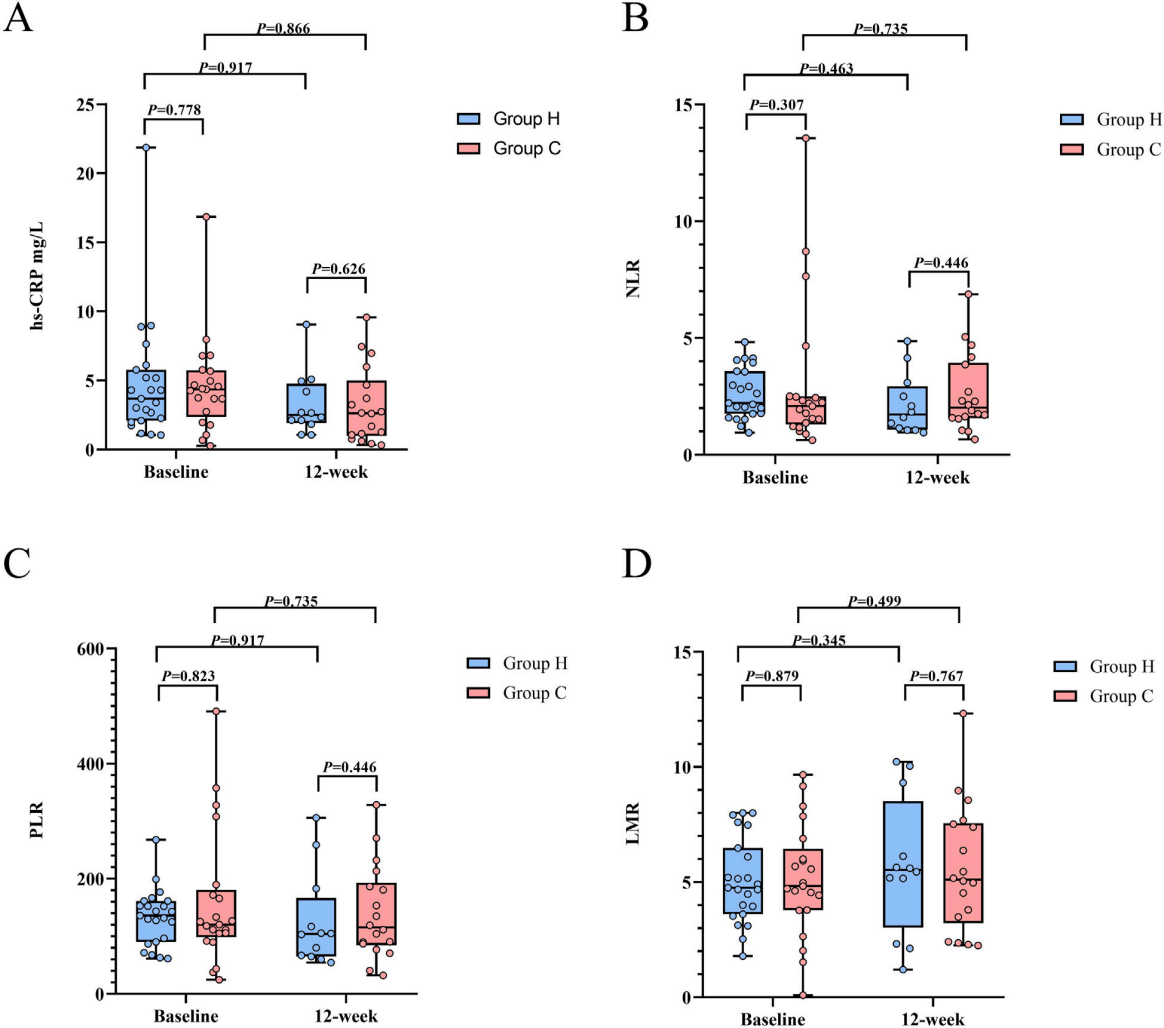
Inflammation levels between Group H and Group C during the study period. **(A)** Distribution of hs-CRP. **(B)** Distribution of NLR. **(C)** Distribution of PLR. **(D)** Distribution of LMR. Abbreviation: hs-CRP, high-sensitivity C-reactive protein; NLR, neutrophil-lymphocyte ratio; PLR, platelet-lymphocyte ratio; LMR, lymphocyte-monocyte ratio.

A total of 23 subjects in Group H and 21 subjects in Group C underwent baseline testing for hs-CRP, NLR, PLR, and LMR. The baseline levels of hs-CRP (Group H: 3.69 [2.09–5.78] vs. Group C: 4.36 [2.36–5.73], Z (42) = −0.282, P = 0.778), NLR (Group H: 2.22 [1.76–3.58] vs. Group C: 2.08 [1.29–2.50], Z (42) = −1.022, P = 0.307), PLR (Group H: 135.9 [90.2–161.5] vs. Group C: 120.6 [98.7–180.8], Z (42) = −0.223, P = 0.823), and LMR (Group H: 4.76 [3.61–6.48] vs. Group C: 4.83 [3.78–6.45], Z (42) = -0.153, P = 0.879) were comparable between the two groups (Table 3; Figure 6). At the trial end, 12 subjects from Group H and 18 subjects from Group C underwent the same tests. Compared to baseline, Group H exhibited reductions in hs-CRP (baseline: 3.69 [2.09–5.78] vs. week 12: 2.50 [1.91–4.77]; Z (5) = −0.105, P = 0.917), NLR (baseline: 2.22 [1.76–3.58] vs. week 12: 1.73 [1.08–2.94]; Z (5) = −0.734, P = 0.463), and PLR (baseline: 135.9 [90.2–161.5] vs. week 12: 104.2 [65.3–166.6]; Z (5) = -0.105, P = 0.917), alongside an increase in LMR (baseline: 4.76 [3.61–6.48] vs. week 12: 5.53 [3.03–8.51]; Z (5) = −0.943, P = 0.345), though none of these changes reached statistical significance. Similarly, in Group C, hs-CRP (baseline: 4.36 [2.36–5.73] vs. week 12: 2.63 [0.99–5.01]; Z (6) = −0.169, P = 0.866), NLR (baseline: 2.08 [1.29–2.50] vs. week 12: 2.02 [1.57–3.95]; Z (6) = -0.338, P = 0.735), and PLR (baseline: 120.6 [98.7–180.8] vs. week 12: 115.4 [84.6–193.3]; Z (6) = -0.338, P = 0.735) decreased, while LMR (baseline: 4.83 [3.78–6.45] vs. week 12: 5.11 [3.22–7.56]; Z (6) = −0.676, P = 0.499) increased, but these changes were also not statistically significant. However, no significant differences were found between the groups in hs-CRP (Group H: 2.50 [1.91–4.77] vs. Group C: 2.63 [0.99–5.00], Z (28) = −0.487, P = 0.626), NLR (Group H: 1.73 [1.08–2.94] vs. Group C: 2.02 [1.57–3.95], Z (28) = −0.762, P = 0.446), PLR (Group H: 104.2 [65.3–166.6] vs. Group C: 115.4 [84.7–193.3], Z (28) = -0.762, P = 0.446), and LMR (Group H: 5.53 [3.03–8.51] vs. Group C: 5.11 [3.22–7.56], Z (28) = -0.296, P = 0.767) at the 12-week point (Table 3; Figure 6). Similar results were observed in the per-protocol analysis (Supplementary Table S3).

**TABLE 3:** ITT analysis of inflammation levels of participants by arm in follow-ups.

Inflammation levels	Group H (n = 23)	Group C (n = 21)	df	Z	P
hs-CRP, median (IQR)					
Baseline	3.69 (2.09–5.78)	4.36 (2.36–5.73)	42	−0.282	0.778
12 weeks	2.50 (1.91–4.77)	2.63 (0.99–5.01)	28	−0.487	0.626
NLR, median (IQR)					
Baseline	2.22 (1.76–3.58)	2.08 (1.29–2.50)	42	−1.022	0.307
12 weeks	1.73 (1.08–2.94)	2.02 (1.57–3.95)	28	−0.762	0.446
PLR, median (IQR)					
Baseline	135.9 (90.2–161.5)	120.6 (98.7–180.8)	42	−0.223	0.823
12 weeks	104.2 (65.3–166.6)	115.4 (84.6–193.3)	28	−0.762	0.446
LMR, median (IQR)					
Baseline	4.76 (3.61–6.48)	4.83 (3.78–6.45)	42	−0.153	0.879
12 weeks	5.53 (3.03–8.51)	5.11 (3.22–7.56)	28	−0.296	0.767

Data are expressed as median (IQR). df = degree of freedom; IQR, interquartile range; hs-CRP , high-sensitivity C-reactive protein; NLR, neutrophil-lymphocyte ratio; PLR, platelet-lymphocyte ratio; LMR, lymphocyte-monocyte ratio.

### 3.5 Quality of life

ITT analysis indicated no significant differences in the scores of PF (Group H: 54.0 ± 15.5 vs. Group C: 51.1 ± 17.8, t (119) = 0.967, P = 0.336), BP (Group H: 42.7 ± 11.3 vs. Group C: 43.4 ± 13.3, t (119) = −0.340, P = 0.735), RP (Group H: 49.6 ± 40.4 vs. Group C: 55.4 ± 39.9, t (119) = −0.798, P = 0.427), RE (Group H: 66.1 ± 39.7 vs. Group C: 70.6 ± 35.3, t (119) = -0.650, P = 0.517), MH (Group H: 66.6 ± 14.0 vs. Group C: 69.0 ± 15.6, t (119) = −0.882, P = 0.380), SF (Group H: 78.5 ± 14.8 vs. Group C: 81.5 ± 16.8, t (119) = -1.032, P = 0.304), VIT (Group H: 65.8 ± 14.3 vs. Group C: 67.5 ± 11.3, t (119) = −0.717, P = 0.475), and GH (Group H: 45.6 ± 15.3 vs. Group C: 47.3 ± 17.8, t (119) = −0.565, P = 0.572) between the two groups at baseline (Table 4). Following the 12-week study period, both groups exhibited significant increases in the scores of PF (Group H: MD −22.0, 95%CI −25.4 to −18.7, t (60) = −13.113, P < 0.001; Group C: MD -21.6, 95%CI −25.1 to 18.0, t (59) = −12.103, P < 0.001), BP (Group H: MD −24.7, 95%CI −27.8 to −21.7, t (60) = −16.109, P < 0.001; Group C: MD −21.4, 95%CI −24.3 to −18.4, t (59) = −14.592, P < 0.001), RP (Group H: MD −36.8, 95%CI −46.4 to −27.3, t (60) = −7.719, P < 0.001; Group C: MD −28.9, 95%CI −37.1 to −20.7, t (59) = -7.083, P < 0.001), RE (Group H: MD −20.9, 95%CI −30.7 to −11.0, t (60) = −4.241, P < 0.001; Group C: MD −22.1, 95%CI -30.9 to −13.4, t (59) = -5.066, P < 0.001), MH (Group H: MD -14.2, 95%CI −17.7 to −10.7, t (60) = −8.050, P < 0.001; Group C: MD −11.5, 95%CI −15.4 to −7.7, t (59) = −5.954, P < 0.001), SF (Group H: MD −21.7, 95%CI −25.3 to −18.1, t (60) = −12.088, P < 0.001; Group C: MD −17.3, 95%CI −21.4 to −13.2, t (59) = −8.368, P < 0.001), VIT (Group H: MD −13.0, 95%CI −16.6 to −9.5, t (60) = −7.330, P < 0.001; Group C: MD −10.0, 95%CI −12.9 to −7.2, t (59) = −7.005, P < 0.001), and GH (Group H: MD −9.5, 95%CI −12.7 to −6.1, t (60) = -5.687, P < 0.001; Group C: MD −7.5, 95%CI -10.8 to −4.2, t (59) = -4.522, P < 0.001) compared to baseline (Supplementary Table S4). However, no significant difference was observed in the scores of PF (Group H: 76.0 ± 12.8 vs. Group C: 72.6 ± 11.7, t (119) = 1.521, P = 0.131), BP (Group H: 67.4 ± 13.7 vs. Group C: 64.8 ± 10.8, t (119) = 1.158, P = 0.249), RP (Group H: 86.4 ± 22.4 vs. Group C: 84.3 ± 21.1, t (119) = 0.532, P = 0.596), RE (Group H: 87.0 ± 20.6 vs. Group C: 92.7 ± 16.3, t (119) = −1.684, P = 0.095), MH (Group H: 80.8 ± 9.5 vs. Group C: 80.5 ± 8.1, t (119) = 0.177, P = 0.860), SF (Group H: 100.2 ± 12.8 vs. Group C: 98.8 ± 12.4, t (119) = 0.629, P = 0.530), VIT (Group H: 78.9 ± 9.0 vs. Group C: 77.5 ± 8.0, t (119) = 0.837, P = 0.404), and GH (Group H: 55.0 ± 15.6 vs. Group C: 54.8 ± 15.7, t (119) = 0.087, P = 0.931) between the two groups at the 12-week (Table 4). Similar findings were observed in the pre-protocol analysis (Supplementary Tables S5, S6).

**TABLE 4:** ITT analysis of SF-36 in baseline and 12-week by arm.

SF-36	Group H (n = 61)	Group C (n = 60)	Mean difference (95% CI)	df	t	P
Physical Function, mean (95% CI)						
Baseline	54.0 (50.0, 58.0)	51.1 (46.5, 55.7)	2.9 (−3.1, 8.9)	119	0.967	0.336
12 weeks	76.0 (72.8, 79.3)	72.6 (69.6, 75.6)	3.4 (−1.0, 7.8)	119	1.521	0.131
Social Function, mean (95% CI)						
Baseline	78.5 (74.7, 82.3)	81.5 (77.1, 85.8)	−3.0 (−8.7, 2.7)	119	−1.032	0.304
12 weeks	100.2 (96.9, 103.5)	98.8 (95.6, 101.9)	1.4 (−3.1, 5.9)	119	0.629	0.530
Physical Role, mean (95% CI)						
Baseline	49.6 (39.2, 59.9)	55.4 (45.1, 65.7)	−5.8 (−20.3, 8.6)	119	−0.798	0.427
12 weeks	86.4 (80.8, 92.1)	84.3 (78.9, 89.8)	2.1 (−5.7, 9.9)	119	0.532	0.596
Emotional Role, mean (95% CI)						
Baseline	66.1 (56.0, 76.3)	70.6 (61.4, 79.7)	−4.4 (−18.0, 9.1)	119	−0.650	0.517
12 weeks	87.0 (81.7, 92.3)	92.7 (88.5, 96.9)	−5.7 (−12.4, 1.0)	119	−1.684	0.095
General Health, mean (95% CI)						
Baseline	45.6 (41.6, 49.5)	47.3 (42.7, 51.9)	−1.7 (−7.7, 4.3)	119	−0.565	0.572
12 weeks	55.0 (51.0, 59.0)	54.8 (50.7, 58.8)	0.2 (−5.4, 5.9)	119	0.087	0.931
Mental Health, mean (95% CI)						
Baseline	66.6 (63.0, 70.2)	69.0 (65.0, 73.0)	−2.4 (−7.7, 3.0)	119	−0.882	0.380
12 weeks	80.8 (78.4, 83.2)	80.5 (78.5, 82.6)	0.3 (−2.9, 3.5)	119	0.177	0.860
Body Pain, mean (95% CI)						
Baseline	42.7 (39.8, 45.5)	43.4 (40.0, 46.9)	−0.8 (−5.2, 3.7)	119	−0.340	0.735
12 weeks	67.4 (63.9, 70.9)	64.8 (62.0, 67.6)	2.6 (−1.8, 7.0)	119	1.158	0.249
Vitality, mean (95% CI)						
Baseline	65.8 (62.1, 69.5)	67.5 (64.6, 70.4)	−1.7 (−6.3, 3.0)	119	−0.717	0.475
12 weeks	78.9 (75.5, 79.6)	77.5 (75.5, 79.6)	1.3 (−1.7, 4.4)	119	0.837	0.404

Data are expressed as mean (95% CI). df = degree of freedom; CI, confidence interval.

### 3.6 Adherence and adverse events

In terms of adherence, ITT analysis revealed no significant difference in the EARS score between the two groups (Group H: 21.5 ± 3.7 vs. Group C: 20.9 ± 3.0, t (119) = 1.046, P = 0.298). A similar finding was observed in pre-protocol analysis (Group H: 21.8 ± 2.6 vs. Group C: 21.5 ± 2.7, t (90) = 0.513, P = 0.609). Regarding adverse events, Group H reported a total of 3 adverse events (5.0%, 3/61), including 2 cases of headache (3.3%, 2/61) and 1 case of nasal cavity dryness (1.6%, 1/61). In contrast, Group C reported 1 adverse event of lower limb muscle pain (1.7%, 1/60). No significant difference was observed in the incidence of adverse events between the two groups (Group H: 5.0%, 3/61 vs. Group C: 1.6%, 1/60, χ^2^ = 0.242, P = 0.619). In addition, we observed that 3 subjects in Group H and 2 subjects in Group C experienced worsening knee-joint pain and subsequently received intra-articular injections. Therefore, these 5 subjects withdrew from the study and were excluded from the pre-protocol analysis.

## 4 Discussion

To the best of our knowledge, it is the first clinical study investigating the effects of molecular hydrogen on symptom improvement in elderly patients with KOA. Our study found that the H_2_-O_2_ mixture inhalation provided improvement in functional status, as measured by the WOMAC, compared to those who did not receive H_2_-O_2_ mixture inhalation in elderly KOA patients undergoing the same HBE program during the 12-week study period. However, the improvement in the WOMAC score was 8 points, which is below the minimum clinically important difference of 9 points. Additionally, there were no difference in other secondary outcomes including inflammation levels, objective function tests, QoL improvement, and adverse events.

Although KOA stands as the primary cause of disability, there are currently no available cures or disease-modifying treatments (Hunter et al., 2020). The degeneration and damage of articular cartilage are considered pivotal mechanisms in the development of KOA, with oxidative stress and inflammatory reaction collectively contributing to this pathological process (Lv et al., 2021; Shumnalieva et al., 2023; Liao et al., 2023). Current research heavily focused on delaying cartilage damage and degeneration. It is well established that reactive oxygen species (ROS) can induce extracellular matrix loss, cell aging, mitochondrial dysfunction, and cell apoptosis in chondrocytes and mesenchymal stem cells, leading to subchondral bone loss and accelerating the occurrence of OA (Tudorachi et al., 2021). Exercise therapy is widely prescribed as a primary treatment for KOA due to its potential to reduce oxidative stress and decrease inflammation (Fransen et al., 2015; Wellsandt and Golightly, 2018). In addition, molecular hydrogen has also been proven to play a significant role in treating ROS-induced diseases and inflammation-related diseases. Ishibashi et al. demonstrated that drinking hydrogen-rich water could reduce the Disease Activity Score in 28 joints (DAS28) and 8-OHdG levels in patients with rheumatic arthritis (RA) (Ishibashi et al., 2012). Another study also indicated that hydrogen-rich saline infusion could decrease DAS28, with significant reductions in serum biomarkers such as IL-6 and 8-OHdG following the infusion (Ishibashi et al., 2014). The underlying mechanism may be attributed to the properties of hydrogen molecules in inhibiting oxidative stress and suppressing inflammatory responses. Increasing evidence suggests that hydrogen molecules can enhance antioxidant capacity by inhibiting the Nrf-2/HO-1 and NF-κB pathways, thereby contributing therapeutically to wound healing, hypoxia/ischemia injury, and Alzheimer’s disease (Li et al., 2022; Cai et al., 2008; Wang et al., 2011). Additionally, molecular hydrogen exerts antioxidant effects by directly neutralizing harmful free radicals, such as hydroxyl radicals (⋅OH) and ONOO⁻, without affecting physiologically important radicals like nitric oxide (Ohta, 2011). Further evidence indicates that hydrogen molecules may counteract the progression of OA by inhibiting oxidative stress, matrix catabolism, and apoptosis (Cheng et al., 2020b). Moreover, prior studies have illustrated that the anti-inflammatory properties of hydrogen encompass the downregulation of various pro-inflammatory cytokines and chemokines, including TNF-α and IL-6, alongside the upregulation of transcription levels of anti-inflammatory cytokines such as IL-10 (Saramago et al., 2019; Nogueira et al., 2018). Reducing inflammatory factors could be beneficial in delaying the progression of KOA and improving the related symptoms.

RA is known to be associated with autoimmune disorders, differing from osteoarthritis (OA), however, both conditions are driven by oxidative stress and inflammatory responses. Consequently, we speculate that the enhancement of KOA symptoms resulting from the combination of H_2_-O_2_ mixture inhalation and home-based exercises was superior to the effects of home-based exercises alone. Despite the observed improvement in WOMAC scores, the combination of H_2_-O_2_ mixture inhalation and home-based exercises did not yield a clinically significant difference at week 12 (MCID: 9-22 on the WOMAC scale) compared to home-based exercises alone. A potential explanation for this finding is that the 2-week inhalation therapy might not have provided sufficient effects of inhibiting oxidative stress and inflammatory responses to achieve a meaningful clinical difference. As indicated by the inflammatory levels, although no significant differences were observed in hs-CRP, NLR, PLR, and LMR between the two groups at week 12, hs-CRP, NLR, and PLR decreased while LMR increased when compared to baseline. This suggested a reduction in inflammation levels, though it has not yet reached statistical significance. We cannot rule out that an extended duration of H_2_-O_2_ mixture inhalation or alternative intervention methods, such as hydrogen-rich saline infusion or injection into the knee joint, might yield different outcomes.

The lack of a clinically significant difference in the improvement of KOA symptoms may be multifactorial. The improvements depend heavily on the adherence to home-based exercise, as well as other factors such as age, sex, KL grade, and duration of KOA (Marks, 2012). In our study, the EARS score was comparable in both groups. Participants with poor adherence to HBE program were excluded from the pre-protocol analysis, yet the primary outcome remained consistent with the ITT analysis. Additionally, the subgroup analysis was performed to identify the effects of other factors including age, sex, KL grade, EARS score, and duration of KOA on clinical outcome (WOMAC score). The results indicated no significant differences in the improvement of KOA symptoms due to H_2_-O_2_ mixture inhalation therapy across different patient groups, particularly among those with differing levels of adherence to HBE program. This underscores the need for further studies focusing on the effects of different methods and durations of hydrogen therapy in alleviating symptoms of KOA.

This study included multiple evaluations at weeks 2, 6, and 12 to examine the sustained effects of H_2_-O_2_ inhalation therapy on symptom improvement in KOA patients. In Hirohisa Ono’s research, a 6-month course of H_2_-O_2_ inhalation significantly improved outcomes assessed by Alzheimer’s Disease Assessment Scale-Cognitive section (ADAS-Cog) and Diffusion Tensor Imaging (DTI), providing not only temporary relief but also sustained benefits for at least 6 months without further H_2_-O_2_ treatment in patients with Alzheimer’s disease (Ono et al., 2023). Similarly, Ishibashi et al. reported that a 5-day hydrogen-saline infusion reduced the disease activity score in 28 joints and levels of IL-6, 8-OHdG, and MMP3 in patients with RA, with effects lasting up to 4 weeks (Ishibashi et al., 2014). Despite the mechanisms remain unclear, these effects may involve anti-cell-death properties, such as ferroptosis inhibition via peroxide reduction and modulation of pro- and anti-death factors (Iuchi et al., 2019; Terasaki et al., 2011). However, our study did not observe sustained symptom improvement in KOA patients following H_2_-O_2_ inhalation therapy. Future research will focus on exploring dose-response relationship of H_2_-O_2_ inhalation therapy to better understand its therapeutic potential.

Regarding the safety of H_2_-O_2_ mixture inhalation, our study observed adverse reactions of headache and dryness of the nasal cavity, which spontaneously resolved upon discontinuation of H_2_-O_2_ inhalation. Headache may be related to vasodilation caused by hydrogen molecules, as a study reported that H_2_-O_2_ mixture inhalation could reduce levels of plasma angiotensin II and aldosterone, thus dilating blood vessels and lowering blood pressure (Liu et al., 2022). Dryness of the nasal cavity may be attributed to the inhalation method through nasal tubes and the flow rate of inhalation (Chen J. B. et al., 2019). Besides, lots of published clinical trials involving the inhalation of gas composed of approximately 67% H_2_ and 33% O_2_ have not reported any severe adverse events (Lin et al., 2020; Dan et al., 2023; Zheng et al., 2021).

Limitations remain in our study. Firstly, this open-label trial implies that both researchers and subjects are aware of the actual intervention measures, which may lead to potential bias in patient-reported outcome data. Secondly, we employed various methods, including health lectures, telephone follow-ups, and phased rewards, to enhance participants’ adherence with home-based exercises, and no differences in EARS scores were found between the two groups. However, information bias may still be unavoidable since the adherence evaluation was conducted at the end of the trial and relied on a retrospective assessment of self-reported home-based exercises. Thirdly, this study did not analyze the levels of plasma inflammatory markers including IL-6, IL-10 and TNF-α, nor did it conduct relevant animal and cell experiments to explore the mechanisms by which hydrogen molecules improve KOA symptoms. Fourth, the control group did not receive O₂ inhalation. According to the formula for calculating the fraction of inspired O₂ (FiO₂ = 21% + 4 × oxygen flow rate), the FiO₂ for the intervention group was approximately 25%, while the control group had an FiO₂ of 21%, corresponding to the ambient air O₂ concentration. Although a 4% difference in FiO₂ is unlikely to have influenced the results and no research has demonstrated that O₂ inhalation had therapeutic effects on OA, it remains a limitation of this study. Finally, the participants living in this CCRC had a high level of education and good health awareness, regularly receiving health guidance from their family physicians. Therefore, we cannot determine whether these results can be extrapolated to other elderly groups.

## 5 Conclusion

As an adjunctive treatment, H_2_-O_2_ mixture inhalation seems to demonstrate therapeutic potential in improving KOA symptoms in elderly patients undergoing HBE program in the first 2 weeks, even though a clinically significant difference was not achieved. Sustained effect of H_2_-O_2_ mixture inhalation in improving KOA symptoms was not observed. Future research should explore varying inhalation durations and intervention methods, alongside *in vitro* and *in vivo* experiments, to further elucidate the role and mechanisms of hydrogen molecules in KOA disease.

## Data Availability

The raw data supporting the conclusions of this article will be made available by the authors, without undue reservation.
