# Supplementary material for: Hydrogen-oxygen mixture inhalation as an adjunctive treatment to home-based exercise in older patients with knee osteoarthritis: an open-label, blinded-endpoint, randomized controlled trial
Source: Front Pharmacol. 2025 Jan 30;16:1505922. doi: 10.3389/fphar.2025.1505922 (PMC11821916; doi:10.3389/fphar.2025.1505922)
Supplement: Supplementary file 1 [file Table1.docx]

Supplementary Material

# Supplementary Tables

Table S1. Pre-protocol analysis of demographic and clinical characteristics of participants by arm in baseline

| Variables | Total (n=92) | Group H (n=46) | Group C (n=46) | df | t, Z or Chi-square | P |
| --- | --- | --- | --- | --- | --- | --- |
| Age, years, mean (SD) | 81.1 (6.2) | 80.6 (5.7) | 81.6 (6.7) | 90 | -0.788 | 0.433 |
| Sex, female, n (%) | 71 (77.2) | 36 (78.3) | 35 (76.1) | 1 | 0.062 | 0.804 |
| BMI, kg/m^2^, mean (SD) | 23.7 (3.0) | 24.1 (2.9) | 23.3 (3.1) | 90 | 1.318 | 0.191 |
| Affected side, n (%) |  |  |  | 1 | 1.181 | 0.277 |
| Unilateral | 33 (35.9) | 19 (41.3) | 14 (30.4) |  |  |  |
| Bilateral | 59 (64.1) | 27 (58.7) | 32 (69.6) |  |  |  |
| KL grade, n (%) |  |  |  | 1 | 1.087 | 0.297 |
| 2 | 47 (51.1) | 21 (45.7) | 26 (56.5) |  |  |  |
| 3 | 45 (48.9) | 25 (54.3) | 20 (43.5) |  |  |  |
| NRS in the past week, mean (SD) | 4.3 (0.7) | 4.4 (0.6) | 4.3 (0.7) | 90 | 0.159 | 0.874 |
| Course of KOA, years, median (IQR) | 17 (10-30) | 20 (10-30) | 15.5 (9.5-30) | 90 | -0.415 | 0.678 |
| Education level, n (%) |  |  |  | 2 | 5.225 | 0.073 |
| High school education or less | 13 (14.1) | 5 (10.9) | 8 (17.4) |  |  |  |
| College degree | 28 (30.4) | 19 (41.3) | 9 (19.6) |  |  |  |
| Bachelor degree or above | 51 (55.4) | 22 (47.8) | 29 (63.0) |  |  |  |
| Cigarette smoking, n (%) | 4 (4.3) | 2 (4.3) | 2 (4.3) | 1 | <0.001 | 1.000 |
| Alcohol drinking, n (%) | 9 (9.8) | 4 (8.7) | 5 (10.9) | 1 | <0.001 | 1.000 |
| Medical history, n (%) |  |  |  |  |  |  |
| Respiratory disease | 28 (30.4) | 14 (30.4) | 14 (30.4) | 1 | <0.001 | 1.000 |
| Hypertension | 68 (73.9) | 38 (82.6) | 30 (65.2) | 1 | 3.608 | 0.058 |
| Coronary heart disease | 43 (46.7) | 22 (47.8) | 21 (45.7) | 1 | 0.044 | 0.834 |
| Gastrointestinal disease | 21 (22.8) | 12 (26.1) | 9 (19.6) | 1 | 0.555 | 0.456 |
| Diabetes mellitus | 29 (31.5) | 16 (34.8) | 13 (28.3) | 1 | 0.453 | 0.501 |
| Anemia | 1 (1.1) | 1 (2.2) | 0 (0) | - | - | - |
| Chronic kidney disease | 4 (4.3) | 1 (2.2) | 3 (6.5) | 1 | 0.261 | 0.617 |
| Cancer | 8 (8.7) | 5 (10.9) | 3 (6.5) | 1 | 0.137 | 0.714 |
| Cerebrovascular disease | 22 (23.9) | 9 (19.6) | 13 (28.3) | 1 | 0.956 | 0.328 |
| Other | 19 (20.7) | 9 (19.6) | 10 (21.7) | 1 | 0.066 | 0.797 |
| Analgesics taken, n (%) | 57 (62.0) | 30 (65.2) | 27 (58.7) | 1 | 0.415 | 0.519 |
| Types of analgesics taken, n (%) |  |  |  | 1 | 0.001 | 0.977 |
| NSAIDS | 36 (63.2) | 19 (63.3) | 17 (63.0) |  |  |  |
| Chinese medicine plaster | 21 (36.8) | 11 (36.7) | 10 (37.0) |  |  |  |

Data are expressed as n (%), mean (SD) or median (IQR). SD=standard deviation; BMI=body mass index; KOA=knee osteoarthritis; IQR=interquartile range; NSAIDS=nonsteroidal anti-inflammatory drugs.

Table S2. Pre-protocol analysis of WOMAC scores, CST and TUG of participants by arm in follow-ups

| Clinical Outcomes | Group H (n=46) | Group C (n=46) | Mean Difference (95% CI) | numerator df | denominator df | F | P |
| --- | --- | --- | --- | --- | --- | --- | --- |
| WOMAC Total Scores, mean (95% CI) | | | | | | | |
| Baseline | 93.6 (88.1, 99.1) | 90.3 (84.9, 95.8) | 3.3 (-4.5, 11.1) | 1 | 100.143 | 0.687 | 0.404 |
| 2 weeks | 84.8 (79.3, 90.3) | 88.8 (83.3, 94.3) | -4.0 (-11.8, 3.7) | 1 | 100.143 | 1.031 | 0.307 |
| 6 weeks | 81.1 (75.6, 86.6) | 83.4 (77.9, 88.9) | -2.3 (-10.1, 5.5) | 1 | 100.143 | 0.332 | 0.561 |
| 12 weeks | 69.2 (63.7, 74.6) | 72.7 (67.2, 78.2) | -3.5 (-11.3, 4.2) | 1 | 100.143 | 0.791 | 0.371 |
| WOMAC Pain Scores, mean (95% CI) | | | | | | | |
| Baseline | 18.5 (17.1, 19.9) | 16.9 (15.5, 18.3) | 1.6 (-0.4, 3.5) | 1 | 123.034 | 2.530 | 0.110 |
| 2 weeks | 15.5 (14.1, 16.9) | 16.1 (14.8, 17.5) | -0.6 (-2.6, 1.3) | 1 | 123.034 | 0.399 | 0.524 |
| 6 weeks | 14.7 (13.3, 16.1) | 14.3 (12.9, 15.7) | 0.4 (-2.4, 1.5) | 1 | 123.034 | 0.171 | 0.676 |
| 12 weeks | 11.9 (10.5, 13.3) | 11.4 (10.1, 12.8) | 0.5 (-1.5, 2.4) | 1 | 123.034 | 0.230 | 0.629 |
| WOMAC Stiffness Scores, mean (95% CI) | | | | | | | |
| Baseline | 9.4 (8.4, 10.4) | 8.1 (7.0, 9.1) | 1.3 (-0.1, 2.8) | 1 | 105.332 | 3.266 | 0.070 |
| 2 weeks | 8.9 (7.9, 9.9) | 8.0 (7.0, 9.1) | 0.9 (-0.6, 2.3) | 1 | 105.332 | 1.359 | 0.241 |
| 6 weeks | 8.6 (7.6, 9.6) | 7.7 (6.7, 8.8) | 0.9 (-0.6, 2.3) | 1 | 105.332 | 1.359 | 0.241 |
| 12 weeks | 7.3 (6.2, 8.3) | 7.1 (6.0, 8.1) | 0.2 (-1.3, 1.7) | 1 | 105.332 | 0.069 | 0.791 |
| WOMAC Function Scores, mean (95% CI) | | | | | | | |
| Baseline | 65.7 (61.0, 70.5) | 65.4 (60.6, 70.2) | 0.3 (-6.4, 7.1) | 1 | 98.397 | 0.010 | 0.919 |
| 2 weeks | 60.4 (55.6, 65.2) | 64.7 (59.9, 69.4) | -4.3 (-11.0, 2.5) | 1 | 98.397 | 1.538 | 0.213 |
| 6 weeks | 57.8 (53.1, 62.6) | 61.4 (56.6, 66.2) | -3.6 (-10.3, 3.2) | 1 | 98.397 | 1.077 | 0.297 |
| 12 weeks | 50.0 (45.2, 54.7) | 54.2 (49.4, 58.9) | -4.2 (-10.9, 2.5) | 1 | 98.397 | 1.491 | 0.220 |
| Chair Stand Test (CST), mean (95% CI) | | | | | | | |
| Baseline | 8.5 (7.6, 9.4) | 8.2 (7.3, 9.1) | 0.3 (-1.0, 1.6) | 1 | 101.768 | 0.208 | 0.645 |
| 2 weeks | 9.0 (8.1, 9.9) | 8.3 (7.4, 9.3) | 0.7 (-0.7, 1.9) | 1 | 101.768 | 0.894 | 0.341 |
| 6 weeks | 10.1 (9.2, 11.1) | 9.4 (8.5, 10.4) | 0.7 (-0.6, 2.0) | 1 | 101.768 | 1.089 | 0.294 |
| 12 weeks | 11.6 (10.7, 12.5) | 10.8 (9.8, 11.7) | 0.8 (-0.5, 2.1) | 1 | 101.768 | 1.535 | 0.213 |
| Timed Up and Go (TUG), mean (95% CI) | | | | | | | |
| Baseline | 14.4 (12.6, 16.1) | 14.9 (13.1, 16.7) | -0.5 (-3.0, 2.0) | 1 | 96.884 | 0.175 | 0.673 |
| 2 weeks | 13.6 (11.9, 15.4) | 14.7 (12.9, 16.5) | -1.1 (-3.6, 1.4) | 1 | 96.884 | 0.682 | 0.406 |
| 6 weeks | 12.8 (11.0, 14.5) | 13.7 (11.9, 15.5) | -0.9 (-3.4, 1.6) | 1 | 96.884 | 0.546 | 0.457 |
| 12 weeks | 12.0 (10.2, 13.8) | 12.8 (11.0, 14.6) | -0.8 (-3.3, 1.7) | 1 | 96.884 | 0.406 | 0.521 |

Data are expressed as mean (95% CI). WOMAC=Western Ontario and McMaster Universities Osteoarthritis Index; CST=chair stand test; TUG=timed up and go; CI=confidence interval.

Notes: WOMAC total score: group× time interaction, F(3, 270)=10.283, P<0.001. WOMAC pain score: group× time interaction, F(3, 270)=4.148, P=0.007. WOMAC stiffness score: group× time interaction, F(3, 270)=3.946, P=0.009. WOMAC function score: group× time interaction, F(3, 270)=6.997, P<0.001. CST: group× time interaction, F(3, 270)=1.380, P=0.249. TUG: group× time interaction, F(3, 270)=0.636, P=0.592.

Table S3. Pre-protocol analysis of inflammation levels of participants by arm in follow-ups

| Inflammation levels | Group H (n=17) | Group C (n=17) | df | Z | P |
| --- | --- | --- | --- | --- | --- |
| hs-CRP, median (IQR) | | | | | |
| Baseline | 4.30 (1.87-6.42) | 4.41 (2.83-6.29) | 32 | -0.465 | 0.642 |
| 12 weeks | 3.44 (2.29-5.31) | 2.63 (0.73-3.95) | 22 | -1.259 | 0.208 |
| NLR, median (IQR) | | | | | |
| Baseline | 2.01 (1.53-2.96) | 2.21 (1.45-3.58) | 32 | -0.086 | 0.931 |
| 12 weeks | 1.97 (1.06-4.29) | 2.02 (1.57-3.07) | 22 | -0.293 | 0.770 |
| PLR, median (IQR) | | | | | |
| Baseline | 130.1 (69.8-152.6) | 126.9 (108.7-248.7) | 32 | -1.154 | 0.249 |
| 12 weeks | 111.1 (66.4-270.7) | 108.0 (84.7-182.2) | 22 | -0.527 | 0.598 |
| LMR, median (IQR) | | | | | |
| Baseline | 5.20 (4.58-7.76) | 4.62 (3.21-5.97) | 32 | -1.498 | 0.134 |
| 12 weeks | 5.32 (2.27-5.76) | 5.32 (3.72-7.91) | 22 | -0.878 | 0.380 |

Data are expressed as median (IQR). IQR=interquartile range; hs-CRP= high-sensitivity C-reactive protein; NLR=neutrophil-lymphocyte ratio; PLR=platelet-lymphocyte ratio; LMR=lymphocyte-monocyte ratio.

Table S4. ITT analysis of SF-36 changes from baseline to 12-week by arm

| SF-36 | Group H (n=61) | | Mean Difference (95% CI) | df | t | P | Group C (n=60) | | Mean Difference (95% CI) | df | t | P |
| --- | --- | --- | --- | --- | --- | --- | --- | --- | --- | --- | --- | --- |
|  | Baseline, mean (SD) | 12-week, mean (SD) |  |  |  |  | Baseline, mean (SD) | 12-week, mean (SD) |  |  |  |  |
| PF | 54.0 (15.5) | 76.0 (12.8) | -22.0 (-25.4, -18.7) | 60 | -13.113 | <0.001 | 51.1 (17.8) | 72.6 (11.7) | -21.6 (-25.1, 18.0) | 59 | -12.103 | <0.001 |
| SF | 78.5 (14.8) | 100.2 (12.8) | -21.7 (-25.3, -18.1) | 60 | -12.088 | <0.001 | 81.5 (16.8) | 98.8 (12.4) | -17.3 (-21.4, -13.2) | 59 | -8.368 | <0.001 |
| RP | 49.6 (40.4) | 86.4 (22.4) | -36.8 (-46.4, -27.3) | 60 | -7.719 | <0.001 | 55.4 (39.9) | 84.3 (21.1) | -28.9 (-37.1, -20.7) | 59 | -7.083 | <0.001 |
| RE | 66.1 (39.7) | 87.0 (20.6) | -20.9 (-30.7, -11.0) | 60 | -4.241 | <0.001 | 70.6 (35.3) | 92.7 (16.3) | -22.1 (-30.9, -13.4) | 59 | -5.066 | <0.001 |
| GH | 45.6 (15.3) | 55.0 (15.6) | -9.5 (-12.7, -6.1) | 60 | -5.687 | <0.001 | 47.3 (17.8) | 54.8 (15.7) | -7.5 (-10.8, -4.2) | 59 | -4.522 | <0.001 |
| MH | 66.6 (14.0) | 80.8(9.8) | -14.2 (-17.7, -10.7) | 60 | -8.050 | <0.001 | 69.0 (15.6) | 80.5 (8.8) | -11.5 (-15.4, -7.7) | 59 | -5.954 | <0.001 |
| BP | 42.7 (11.3) | 67.4 (13.7) | -24.7 (-27.8, -21.7) | 60 | -16.109 | <0.001 | 43.4 (13.3) | 64.8 (10.8) | -21.4 (-24.3, -18.4) | 59 | -14.592 | <0.001 |
| VIT | 65.8 (14.3) | 78.9(9.0) | -13.0 (-16.6, -9.5) | 60 | -7.330 | <0.001 | 67.5 (11.3) | 77.5 (8.0) | -10.0 (-12.9, -7.2) | 59 | -7.005 | <0.001 |

Data are expressed as mean (SD). SD=standard deviation; CI=confidence interval; PF=Physical Function; BP=Bodily Pain; RP=Role Limitations due to Physical Health Problems; RE=Role Limitations due to Emotional Problems; MH=General Mental Health; SF=Social Function; VIT= Vitality; GH=General Health.

Table S5. Pre-protocol analysis of SF-36 changes from baseline to 12-week by arm

| SF-36 | Group H (n=46) | | Mean Difference (95% CI) | df | t | P | Group C (n=46) | | Mean Difference (95% CI) | df | t | P |
| --- | --- | --- | --- | --- | --- | --- | --- | --- | --- | --- | --- | --- |
|  | Baseline, mean (SD) | 12-week, mean (SD) |  |  |  |  | Baseline, mean (SD) | 12-week, mean (SD) |  |  |  |  |
| PF | 52.4 (15.8) | 75.7 (12.2) | -23.3 (-27.5, -19.0) | 45 | -11.087 | <0.001 | 56.8 (16.6) | 74.0 (12.5) | -17.2 (-20.3, -14.1) | 45 | -11.240 | <0.001 |
| SF | 79.6 (13.8) | 100.5 (13.4) | -20.9 (-25.3, -16.6) | 45 | -9.664 | <0.001 | 83.2 (16.7) | 98.4 (12.8) | -15.2 (-19.8, -10.7) | 45 | -6.725 | <0.001 |
| RP | 48.4 (37.8) | 84.8 (25.5) | -36.4 (-47.2, -25.6) | 45 | -6.784 | <0.001 | 63.0 (40.0) | 88.0 (20.2) | -25.0 (-33.7, -16.3) | 45 | -5.778 | <0.001 |
| RE | 66.7 (39.1) | 88.1 (21.3) | -21.4 (-33.1, -9.8) | 45 | -3.700 | 0.001 | 76.1 (34.9) | 93.5 (17.7) | -17.4 (-27.2, -7.7) | 45 | -3.587 | 0.001 |
| GH | 44.2 (14.3) | 54.5 (16.1) | -10.3 (-13.9, -6.7) | 45 | -5.761 | <0.001 | 50.7 (19.4) | 55.4 (17.5) | -4.7 (-8.2, -1.1) | 45 | -2.695 | 0.010 |
| MH | 67.7 (14.5) | 81.8 (9.1) | -14.2 (-18.3, -10.1) | 45 | -6.994 | <0.001 | 72.5 (12.9) | 80.7 (8.4) | -8.2 (-11.4, -5.0) | 45 | -5.134 | <0.001 |
| BP | 43.3 (10.4) | 67.1 (13.0) | -23.7 (-27.0, -20.4) | 45 | -14.535 | <0.001 | 45.5 (12.7) | 66.0 (11.0) | -20.5 (-23.6, -17.5) | 45 | -13.588 | <0.001 |
| VIT | 66.7 (14.4) | 78.9 (9.5) | -12.2 (-16.5, -7.9) | 45 | -5.718 | <0.001 | 69.3 (10.8) | 78.4 (8.6) | -9.0 (-12.3, -5.8) | 45 | -5.622 | <0.001 |

Data are expressed as mean (SD). SD=standard deviation; CI=confidence interval; PF=Physical Function; BP=Bodily Pain; RP=Role Limitations due to Physical Health Problems; RE=Role Limitations due to Emotional Problems; MH=General Mental Health; SF=Social Function; VIT= Vitality; GH=General Health.

Table S6. Pre-protocol analysis of SF-36 of participants by arm in follow-ups

| SF-36 | Group H (n=46) | Group C (n=46) | Mean Difference (95% CI) | df | t | P |
| --- | --- | --- | --- | --- | --- | --- |
| Physical Function, mean (95% CI) | | | | | | |
| Baseline | 52.4 (47.7, 57.1) | 56.8 (51.9, 61.8) | 4.4 (-2.3, 11.2) | 90 | -1.320 | 0.190 |
| 12 weeks | 75.7 (72.0, 79.3) | 74.0 (70.3, 77.7) | -1.6 (-6.7, 3.5) | 90 | 0.635 | 0.527 |
| Social Function, mean (95% CI) | | | | | | |
| Baseline | 79.6 (75.5, 83.7) | 83.2 (78.2, 88.1) | 3.5 (-2.8, 9.9) | 90 | -1.106 | 0.272 |
| 12 weeks | 100.5 (96.6, 104.5) | 98.4 (94.6, 102.2) | -2.2 (-7.6, 3.3) | 90 | 0.795 | 0.429 |
| Physical Role, mean (95% CI) | | | | | | |
| Baseline | 48.4 (37.1, 59.6) | 63.0 (51.2, 74.9) | 14.7 (-1.5, 30.8) | 90 | -1.808 | 0.074 |
| 12 weeks | 84.8 (77.2, 92.4) | 88.0 (82.0, 94.1) | 3.3 (-6.3, 12.8) | 90 | -0.679 | 0.499 |
| Emotional Role, mean (95% CI) | | | | | | |
| Baseline | 66.7 (55.0, 78.3) | 76.1 (65.7, 86.5) | 9.4 (-5.9, 24.8) | 90 | -1.219 | 0.226 |
| 12 weeks | 88.1 (81.8, 94.4) | 93.5 (88.3, 98.8) | 5.5 (-2.6, 13.6) | 90 | -1.339 | 0.184 |
| General Health, mean (95% CI) | | | | | | |
| Baseline | 44.2 (39.9, 48.4) | 50.7 (44.9, 56.4) | 6.5 (-0.5, 13.6) | 90 | -1.843 | 0.069 |
| 12 weeks | 54.5 (49.7, 59.3) | 55.4 (50.2, 60.6) | 0.9 (-6.1, 7.9) | 90 | -0.254 | 0.800 |
| Mental Health, mean (95% CI) | | | | | | |
| Baseline | 67.6 (63.3, 72.0) | 72.5 (68.7, 76.3) | 4.9 (-0.8, 10.5) | 90 | -1.704 | 0.092 |
| 12 weeks | 81.8 (79.1, 84.5) | 80.7 (78.2, 83.2) | -1.1 (-4.7, 2.5) | 90 | 0.621 | 0.536 |
| Body Pain, mean (95% CI) | | | | | | |
| Baseline | 43.3 (40.2, 46.4) | 45.5 (41.7, 49.3) | 2.2 (-2.6, 7.0) | 90 | -0.900 | 0.371 |
| 12 weeks | 67.1 (63.2, 70.9) | 66.0 (62.8, 69.3) | 2.5 (-6.0, 3.9) | 90 | 0.413 | 0.681 |
| Vitality, mean (95% CI) | | | | | | |
| Baseline | 66.7 (62.5, 71.0) | 69.4 (66.1, 72.6) | 2.6 (-2.7, 7.9) | 90 | -0.983 | 0.329 |
| 12 weeks | 78.9 (76.1, 81.7) | 78.4 (75.8, 80.9) | -0.5 (-4.3, 3.2) | 90 | 0.287 | 0.774 |

Data are expressed as mean (95% CI). CI=confidence interval.

# Supplementary_File_1

**Details of Home-based Knee Exercises**

|  | Details |
| --- | --- |
| Warm-up | Walking for 20-30 minutes. |
| Passive knee flexion | 1. Sit on the bed.  2. Place your hands on one side of the ankle and slowly and forcefully bring the leg toward your chest to achieve maximum knee flexion. Hold this position for 60 seconds.  3. Relax this leg and repeat the same action on the other side.  4. Repeat the exercise 3 times with both legs. |
| Passive knee extension | 1. Sit on the bed with one foot placed on a foot pad elevated 8~10cm.  2. Apply light weight to the raised knee joint or apply proper pressure by hand for 60 seconds.  3. Relax this leg and repeat the same action on the other side.  4. Repeat the exercise 2 times with both legs. |
| Isometric contractions of the quadriceps | 1. Sitting or lying down with your legs relaxed.  2. Contract the thigh muscles on one side as strongly as possible, holding for 5 seconds, then relax for 2 seconds. Repeat this sequence 10 times for one set, and perform 10 sets consecutively.  3. Relax this leg and repeat the same action on the other side.  4. Repeat the exercise 4 times with both legs. |
| Supine straight-leg lifts | 1. Lie on your back with your knees extended.  2. Bend one leg to support the bed surface while raising the other leg towards the heel, approximately 20 cm away from the bed. Hold this position for 5 seconds, then put down for 5 seconds, repeat 10 times.  3. Relax this leg and repeat the same act ion on the other side.  4. Repeat the exercise 3 times with both legs. |
| Leg lifts in the prone position | 1. Begin by lying face down with your knees fully extended.  2. Lift one leg backward towards the toe, approximately 20 cm away from the bed. Hold this position for 5 seconds, then put down for 5 seconds, repeat 10 times.  3. Relax this leg and repeat the same action on the other side.  4. Repeat the exercise 3 times with both legs. |
| Resistance knee extension | 1. Sit on the chair or at the bed, tie a 1kg sandbag to the ankle, keep the upper body straight.  2. Do not move the thighs, lift your calves until the knees are fully extended, hold for 5 seconds, rest your legs for 5 seconds, repeat 10 times.  3. Relax this leg and repeat the above action on the other side.  4. Repeat the exercise 3 times with both legs. |
| Resistance knee flexion | 1. Standing up, tie a 1kg weight sandbag to the ankle joint, and support the upper edge of the chair.  2. Stand on one leg and pull the calf back to the other leg, flexing the knee as much as possible while keeping the thigh perpendicular to the ground. Hold for 5 seconds, put your legs down for 5 seconds, repeat 10 times.  3. Relax this leg and repeat the above action on the other side.  4. Repeat the exercise 3 times with both legs |
